# Epigenetic insights into minimal residual disease detection in cancer

**DOI:** 10.1016/j.gendis.2025.101830

**Published:** 2025-08-22

**Authors:** Lingao Ju, Gang Wang, Zilin Xu, Wan Xiang, Hongwei Peng, Mengxue Yu, Shenjuan Li, Yi Zhang, Kaiyu Qian, Yu Xiao

**Affiliations:** a Department of Biological Repositories, Human Genetic Resources Preservation Center of Hubei Province, Zhongnan Hospital of Wuhan University, Wuhan, Hubei 430071, China; b Hubei Key Laboratory of Urological Diseases, Laboratory of Precision Medicine, Zhongnan Hospital of Wuhan University, Wuhan, Hubei 430071, China; c Department of Urology, Zhongnan Hospital of Wuhan University, Wuhan, Hubei 430071, China; d Department of Laboratory Medicine, Zhongnan Hospital of Wuhan University, Wuhan, Hubei, 430071, China; e Euler Technology, ZGC Life Sciences Park, Beijing 102206, China; f High Performance Computing Center, The Peking-Tsinghua College of Life Sciences, Peking University, Beijing 100091, China

The landscape of cancer diagnostics has been fundamentally transformed by the emergence of minimal residual disease (MRD) detection technologies, with epigenetic mechanisms playing a pivotal role in understanding the molecular complexity of cancer.^1^ Epigenetic modifications, particularly DNA methylation (DNAm) and chromatin accessibility (ChrAcc), have emerged as critical determinants in tracking and comprehending residual cancer cells that persist after primary treatment.^2^ DNAm represents a sophisticated molecular mechanism through which cancer cells maintain their adaptive state, enabling MRD detection with unprecedented precision.^3^ Recent single-cell sequencing technologies have revealed that methylation patterns serve as dynamic molecular signatures that can distinguish between active and dormant cancer cell populations.^4^ These epigenetic modifications act as molecular switches, regulating gene expression and cellular plasticity without altering the underlying genetic code, thus providing a nuanced understanding of cancer cell behavior beyond traditional genetic analysis.^5^ ChrAcc, another crucial epigenetic dimension, offers complementary insights into MRD detection. By examining the openness of chromatin regions, specific genomic loci that remain accessible in residual cancer cells can be identified, even after extensive treatment.^2^ This approach allows the identification of rare cancer cell populations that might otherwise escape conventional detection methods (Fig. 1A).

**Figure 1:**
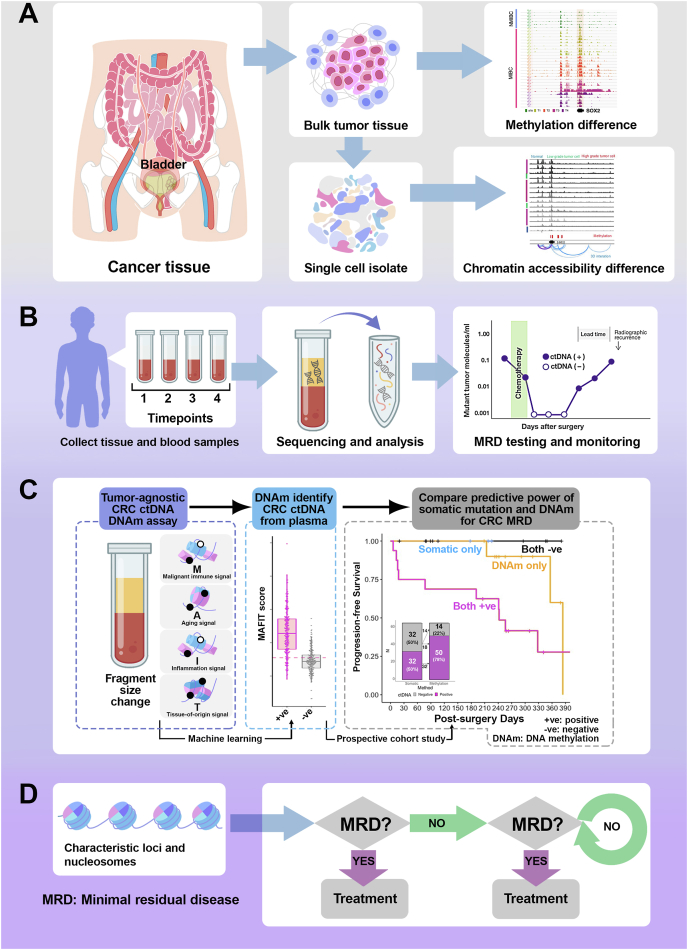
Workflow for minimal residual disease (MRD) detection and personalized clinical management. This schematic illustrates an innovative molecular diagnostic approach for cancer management, integrating advanced technologies from tissue sampling to personalized treatment strategies. **(A)** The workflow systematically progresses through critical stages of MRD detection, beginning with cancer tissue collection and single-cell isolation, followed by sophisticated molecular characterization, including methylation difference and chromatin accessibility analyses. **(B)** Next-generation sequencing and nucleosome analysis enable statistical probability assessment to infer residual tumor content and predict patient risk. The decision-making process is dynamic, with follow-up blood samples collected at multiple timepoints post-surgery to monitor potential disease recurrence. **(C)** Cell-free DNA methylation signature-based MRD detection provides high sensitivity in the surveillance of colorectal cancer recurrence. Cell-free DNA methylation enables precise patient risk stratification, differentiating cohorts with statistically significant recurrence probabilities. Post-surgical blood-based DNA methylation signatures demonstrate exceptional sensitivity in detecting residual disease, providing clinicians with a powerful tool for designing personalized follow-up protocols and determining optimal monitoring intervals. By integrating sophisticated molecular profiling techniques, this approach represents a significant advancement in precision oncology, offering a nuanced framework for individualized colorectal cancer management that transcends traditional diagnostic limitations. **(D)** Based on MRD detection results, clinicians can promptly design targeted interventions, such as chemotherapy, targeted drug therapy, adjuvant radiotherapy, and immunotherapy. The MRD detection approach provides a flexible framework for personalized treatment, allowing for immediate intervention or second-line treatment strategies contingent upon continuous molecular surveillance. This approach represents a paradigm shift in cancer management, emphasizing real-time molecular monitoring and precision therapeutic selection.

The current commentary study of our recent paper regarding the epigenetic role of MRD detection in colorectal cancer^3^ and bladder cancer^4^ enables us to unravel the intricate interplay of DNAm, ChrAcc, and epigenetic reprogramming, creating a complex regulatory landscape that determines cellular fate and the potential for cancer recurrence.

In bladder cancer, the transmembrane-4 L-six family member-1 (TM4SF1)-positive cancer subpopulation represents a paradigmatic example of how epigenetic plasticity contributes to MRD persistence.^4^ These stem-cell-like cells demonstrate remarkable adaptability, generating heterogeneous descendant lineages through epigenetic reprogramming rather than genetic mutation. Such populations can remain dormant and undetectable, representing a significant challenge in comprehensive cancer treatment and monitoring. Emerging technologies, such as single-cell RNA sequencing, T cell receptor sequencing, and ChrAcc sequencing, have revolutionized our understanding of MRD. These techniques enable researchers to profile cancer cells at an unprecedented resolution, revealing molecular trajectories and interactions within the tumor microenvironment. By mapping epigenetic landscapes at the single-cell level, residual disease can now be detected and characterized with remarkable sensitivity.

The clinical implications of advanced MRD detection are profound. Precise identification of residual cancer cells allows for more personalized and adaptive treatment strategies. Clinicians can now monitor treatment response in real-time, adjusting therapeutic approaches on the basis of the molecular characteristics of detected residual populations (Fig. 1B). This represents a paradigm shift from traditional one-size-fits-all treatment models to more nuanced, dynamic, patient-specific intervention strategies.

On the other hand, colorectal cancer represents a significant global health challenge. Recent advancements in molecular diagnostics have revolutionized our understanding of MRD detection, particularly through innovative cell-free DNA analysis techniques that transcend traditional diagnostic limitations. Our recent study revealed a sophisticated approach to cancer detection utilizing DNAm signatures, offering unprecedented sensitivity in identifying the presence of colorectal cancer and predicting recurrence, based on a multi-center, prospective, observational cohort study.^3^ By integrating multiple molecular markers, including tumor-derived DNAm, immune-related methylation signals, age-related methylation patterns, and cell-free DNA fragment size information, we developed a comprehensive metric for assessing tumor-associated molecular changes, named MAFIT score (Fig. 1C). Key findings demonstrate remarkable improvements over conventional somatic mutation detection methods. While traditional mutation analysis identified circulating tumor DNA in 50% of pre-surgical samples, DNAm-based techniques successfully detected tumor signals in 78.1% of cases, revealing significant diagnostic potential. The strength of this methodology lies in its ability to detect cancer-specific molecular signatures independent of individual tumor mutation profiles, effectively addressing limitations in existing screening technologies. The study’s prospective, multi-center cohort design involved 280 colorectal cancer patients across various disease stages, providing robust statistical validation. Notably, post-surgical cell-free DNA analysis revealed that patients with negative DNAm signatures exhibited 100% recurrence-free survival during the observation period, highlighting the technique’s predictive accuracy. By stratifying patients into risk categories based on their MAFIT scores, researchers could differentiate low-, medium-, and high-risk groups with statistically significant progression-free survival variations. Mechanistically, the DNAm approach offers multiple advantages: it normalizes tumor-specific signals, accommodates tumors lacking canonical mutations, and incorporates immune-cell-associated methylation patterns. This comprehensive molecular profiling enables more sensitive and specific MRD detection compared with the traditional mutation-based methods. As molecular diagnostics continue evolving, this DNAm-based MRD detection method represents a significant leap forward in cancer management. Its ability to provide sensitive, specific, and non-invasive tumor monitoring promises to transform clinical practice, offering hope for more personalized and proactive cancer care strategies.

The future of MRD detection lies in integrative approaches that combine multiple molecular profiling techniques (Fig. 1D). By synthesizing data from genomic, transcriptomic, and epigenetic analyses, researchers can develop more comprehensive and precise detection methodologies. Machine learning and artificial intelligence will likely play increasingly important roles in interpreting these complex datasets, enabling more accurate prediction and monitoring of residual disease. Challenges remain in developing universally applicable MRD detection protocols, particularly for low-grade or early-stage cancers. The heterogeneity of cancer types and individual patient variations necessitates continued research and technological innovation.^4^ However, the rapid advancements in single-cell technologies and epigenetic profiling offer unprecedented opportunities for more refined and personalized cancer monitoring.

Taken together, this commentary analysis of our recently published paper regarding MRD in colorectal cancer^3^ and bladder cancer^4^ indicates the integration of epigenetic insights promising for transforming cancer diagnostics and treatment. By deciphering the molecular mechanisms underlying residual disease, researchers are moving closer to developing highly sensitive, non-invasive diagnostic tools that can preemptively identify recurrence risks and guide targeted therapeutic strategies. The integration of DNAm and ChrAcc profiling represents a paradigm shift in precision oncology, promising improvements in patient outcomes and redefining cancer management in the era of molecular medicine.

## Ethics declaration

All research procedures were approved by the Institutional Review Board of the Zhongnan Hospital of Wuhan University (approval number: 2017038-1) and conducted in accordance with the Declaration of Helsinki.

## CRediT authorship contribution statement

**Lingao Ju:** Data curation, Methodology, Investigation, Writing – original draft, Formal analysis, Validation. **Gang Wang:** Methodology, Writing – review & editing, Investigation, Validation, Data curation. **Zilin Xu:** Validation, Investigation. **Wan Xiang:** Resources. **Hongwei Peng:** Resources. **Mengxue Yu:** Investigation. **Shenjuan Li:** Resources. **Yi Zhang:** Data curation, Formal analysis, Software. **Kaiyu Qian:** Methodology, Conceptualization, Writing – review & editing, Funding acquisition, Supervision, Resources. **Yu Xiao:** Methodology, Supervision, Project administration, Conceptualization, Writing – original draft, Funding acquisition.

## Data availability

The sequencing data of the human biospecimens used in this study were derived from our previous study.^3^^,^^4^ The raw sequencing data and clinical information are unique to an individual and require controlled access. The deposited and publicly available data are compliant with the regulations of the Human Genetic Resources Management Office, Ministry of Science and Technology of China (approval number: 2022BAT0129).

## Funding

This study was supported by the 10.13039/501100001809National Natural Science Foundation of China (No. 82372654) and the Research Fund of 10.13039/501100016359Zhongnan Hospital of Wuhan University (China) (No. RLYC2024001001, PTPP2024001). The funders played no role in the study design, data collection and analysis, decision to publish, or preparation of the manuscript.

## Conflict of interests

The authors declared no conflict of interests.
